# Emergent features break the rules of crowding

**DOI:** 10.1038/s41598-019-57277-y

**Published:** 2020-01-15

**Authors:** Natalia Melnik, Daniel R. Coates, Bilge Sayim

**Affiliations:** 1 Institute of Psychology, University of Bern, Bern, 3012 Switzerland; 2 College of Optometry, University of Houston, Houston, Texas 77204 USA; 3 SCALab - Sciences Cognitives et Sciences Affectives, CNRS, UMR 9193, University of Lille, Lille, 59000 France

**Keywords:** Object vision, Human behaviour

## Abstract

Crowding is the deleterious influence of surrounding objects (flankers) on target identification. A central rule of crowding is that it is stronger when the target and the flankers are similar. Here, we show in three experiments how emergent features break this rule. Observers identified targets with various emergent features consisting of a pair of adjacent chevrons either pointing in opposite (‘Diamonds’ and ‘Xs’), or the same (both up or down) directions. Targets were flanked by Diamonds or Xs, resulting in conditions with different levels of target-flanker similarity. Despite high target-flanker similarity, Diamonds were identified better than Xs when flanked by Diamonds. Participants’ judgments of target conspicuity, however, showed that Diamonds were not perceived to stand out more strongly from X than Diamond flankers. Next, we asked observers to indicate whether all presented items were identical. We found superior performance with all Diamonds compared to all Xs, indicating that display uniformity judgments benefitted from the emergent features of Diamonds. Our results showed that emergent features and the information content of the entire display strongly modulated crowding. We suggest that conventional crowding rules only hold when target and flankers are artificially constrained to be mutually independent.

## Introduction

Crowding is the interference of clutter (i.e., flankers) with target perception, impeding target identification^1–4^ and altering target appearance^5–7^. Crowding has been shown to follow several rules. One of the central crowding rules is that it depends on the spacing between the target and the flankers: the closer the flankers are to the target, the stronger the crowding (e.g.^1,8,9^). Another central rule of crowding is its dependence on the similarity between the target and the flankers^9–14^. Crowding is usually strong when target-flanker similarity is high, and it is weak when target-flanker similarity is low. For example, low similarity between the target and flankers in regard to color^9–11,15^, contrast polarity^9,12^, depth^9^, and shape^9,13,14,16^ has been shown to decrease crowding compared to high similarity.

Beyond such local similarity, the strength of grouping between the target and the flankers has been shown to strongly affect crowding^17–22^. In particular, when the target groups with the flankers, performance is usually worse compared to when it does not group with the flankers^18–25^. This ‘global’ grouping has been shown to overrule local similarity (e.g.^15,19,20,24^). For example, performance on a vernier target was superior when the vernier was flanked by single lines of opposite contrast polarity compared to multiple lines alternating in contrast polarity despite the same local context (the innermost flanking lines) in both conditions^15^. The strength of (un)grouping of the target from the flankers is usually related to target conspicuity, i.e., the degree to which the target stands out from the flankers: higher target conspicuity was shown to be associated with weaker crowding^21,24,26^ (but see^27^). These studies showed that strong grouping between the target and the flankers usually deteriorates performance; however, target-flanker grouping can also be beneficial (e.g.^28–31^). For example, grouping between the target and a remote item identical to the target presented outside the crowding zone (in the fovea), improved performance compared to a remote item that was different^28^. A similar improvement due to target-flanker grouping has been recently shown in the crowding zone: strong grouping between the target and a flanker was beneficial when they formed a good Gestalt^29^, showing configural (or object-) superiority effects^31,32^ of grouping in crowding. The benefits of target-flanker grouping in these studies suggest that task-relevant information can be extracted from target-flanker configurations, and that observers may use this information to perform the task. Most crowding rules, however, are based on studies in which target-flanker relations were uninformative, resulting in a monotonic change of performance as a function of the variables at hand, such as target-flanker spacing, similarity, and grouping.

By contrast, in ecologically valid situations, a target’s context is often informative about its identity^33–40^. Here, we investigated what information could be used by observers in a crowding task when multiple sources of information were available. Specifically, we tested if the similarity rule in crowding holds when emergent features and target-flanker relations on different levels of perceptual organization were informative about target identity. Targets consisted of two parts; each part of the target individually, and the emergent features resulting from the grouping of the two parts into larger wholes, provided cues about target identity. We varied the extent to which the target grouped with the flankers by using flankers with either the same or different emergent features as the target. As noted above, strong target-flanker grouping is usually detrimental in crowding; however, as strong grouping is generally associated with higher similarity of the displayed items on a given dimension compared to similar displays with weaker grouping, the higher similarity itself, that is the uniformity of the display, is a potential source of information about the target’s identity^41–44^. In particular, as no item stands out in uniform displays, correctly identifying a flanker may be sufficient to correctly inferring that the target is of the same identity^41,45^.

**Figure 1:**
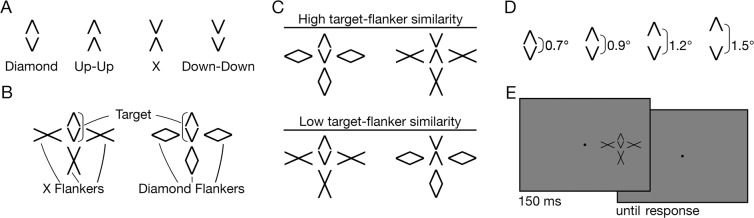
Illustration of the stimuli and task. (**A**) Targets: Illustrations of the four configurations of chevrons. (**B**) A Diamond target flanked by X (left panel) and Diamond (right panel) flankers. (**C**) Stimulus examples with high and low target-flanker similarity. (**D**) The target chevron distance was varied (four levels). (**E**) Time course of a trial (stimuli not shown to scale).

To investigate if emergent features and multiple sources of target information would override the similarity rule of crowding, participants performed three different tasks on the same stimuli. In Experiment 1, observers performed a 4-alternative identification task. Targets were composed of two chevrons, each of which was either pointing up or down, yielding four different configurations that we call “Diamond”, “Up-Up”, “Down-Down”, and “X” (Fig. 1A). These configurations varied in terms of configural information, containing various emergent features such as closure (Diamonds), collinearity (Xs), and translational symmetry (Up-Up and Down-Down). We varied target-flanker similarity, defined as the similarity of target and flanker shapes. There were two types of flankers, Diamonds and Xs, consisting of the same chevrons as the targets (Fig. 1B). Target-flanker similarity was high when the target and flanker configurations were the same, for example, when a Diamond target was flanked by Diamonds (Fig. 1C). Target-flanker similarity was low when the target and the flanker configurations were different, for example, when an X target was flanked by Diamonds. Hence, there were two levels of shape similarity (low and high). We also modulated the similarity between the targets and flankers by varying the distance between the two target chevrons (Fig. 1D). As the highest physical similarity of two shapes with identical parts occurs when the parts are the same distance apart in each shape, similarity between the same shapes was reduced with increasing target chevron distance. Any influence of similarity on target identification was expected to be most pronounced at the closest chevron spacing where flankers were – except for a small vertical translation - exact copies of the corresponding targets. Target configurations and chevron distances were considered separately without assumptions about changes of similarity resulting from their interactions.

Contrary to the typical effect of target-flanker similarity, we found that performance with the Diamond targets was better compared to performance with the X targets, both when flanked by Diamonds or Xs, breaking the similarity rule of crowding. Usually, better performance in crowding positively correlates with target conspicuity. To test if the results could be attributed to differences in target conspicuity, we presented participants with the same stimuli as in Experiment 1 and asked them to judge the conspicuity of the targets (Experiment 2). In short, Diamond targets flanked by Diamonds did not stand out more than X targets flanked by Xs. Hence, it seems that target conspicuity did not underlie the findings of the first experiment. In Experiment 3, we investigated whether the uniformity of the display provided target information that could have benefitted Diamond performance in Experiment 1. Observers judged the uniformity of the display (target and flankers) by indicating whether all presented items were the same or not. Observers were more accurate in reporting the uniformity of the displays with Diamond targets among Diamond flankers compared to X targets among X flankers. This result showed that even when all parts in a display (i.e., the chevrons that composed the target and the flankers) were the same, uniformity judgments varied depending on the arrangements of the parts. We suggest that display uniformity played a role for the superior performance with Diamonds in Experiment 1. Our results showed how a well-established rule of crowding did not hold when target-flanker relations were informative. Observers seem to incorporate sources other than purely local target information when task-relevant information is available in the display as a whole.

## Experiment 1: Configuration Identification

In Experiment 1, observers were presented with targets consisting of a pair of chevrons either pointing in opposite (X and Diamond), or the same (Up-Up and Down-Down) directions (Fig. 1A). The task was to identify the target. Targets were presented among similar (e.g., Diamond flanked by Diamonds; high target-flanker similarity) or dissimilar (e.g., X flanked by Diamonds; low target-flanker similarity) flankers (Fig. 1C). The distance between the target chevrons was varied (Fig. 1D). If target-flanker similarity alone determined crowding, observers’ performance would be worse with high compared to low target-flanker similarity. Alternatively, configural relations between the parts of the target (e.g., its good Gestalt) could determine performance. Relatively high performance would be expected with Diamonds compared to the other targets regardless of the type of flankers if the good Gestalts of Diamonds overcame the effect of target-flanker similarity.

### Methods

#### Observers

Five observers (all females; age range = 22–26) participated in Experiment 1. The sample size was selected based on a study with a similar design^29^. All observers reported normal or corrected to normal vision. They were unaware of the purpose of the experiment. One additional observer was excluded because of reported excessive guessing that resulted in near chance performance. All experiments were carried out with regards to the ethical standards of the Declaration of Helsinki and were approved by the local Ethics Committee of the University of Bern. All recruited observers provided informed consent prior to their participation.

#### Apparatus

Stimuli were presented on a 21-inch CRT color monitor, set at a resolution of 1,024 × 768 pixels and a refresh rate of 120 Hz. A chin- and headrest, positioned at 57 cm from the screen, was used to stabilize the viewing position and secure equal viewing distance among all participants. Python 2.7 and the PsychoPy toolbox^46^ were used for stimulus presentation and data collection. Experiments took place in a dimly lit room. All displayed stimuli were black (0.89 cd/m^2^), presented on a gray (33.3 cd/m^2^) background.

#### Stimuli

Stimuli consisted of configurations formed by two vertically-stacked chevrons positioned at four distances from each other (center-to-center: 0.7°, 0.9°, 1.2°, and 1.5°; Fig. 1D). The lower chevron was positioned at 8° eccentricity in the right visual field and centrally aligned with the fixation point. Configurations of chevrons formed 4 targets: Up-Up (both chevrons pointing up), Down-Down (both chevrons pointing down), Diamond (the lower chevron pointing down, the upper chevron pointing up), and X (the lower chevron pointing up, the upper chevron pointing down; Fig. 1A). The two lines making up the chevrons were 0.67° long and positioned at an angle of 53.13° (chevrons were fitted into a 0.6° × 0.6° bounding box). The lower target chevron was flanked by 3 items located to the right, left, and below (Fig. 1B). The upper target chevron was not surrounded by flankers (i.e., was unflanked). The center-to-center distance between the lower part of the target and the flankers was 1.2° (edge-to-edge distance was 0.3°). There were two types of flankers, “Diamonds” and “Xs”, formed by the same chevrons as used in the targets, but with no gap between the chevrons (Fig. 1B). We refer to Diamond targets flanked by Diamonds and X targets flanked by Xs as the *High target-flanker similarity* (HS) condition; and Diamond targets flanked by Xs and X targets flanked by Diamonds as the *Low target-flanker similarity* (LS) condition. Target-flanker similarity for the Up-Up and Down-Down targets was not varied explicitly.

#### Procedure

Diamond and X flankers were presented in separate blocks. The four targets were counterbalanced within a block and presented in random order. In each block, one spacing between the two target chevrons was used. Stimuli were shown for 150 ms (Fig. 1E), with a 500 ms inter-trial interval after each response. Observers indicated the target by pressing one of the four designated keys on the keyboard. A high-pitched sound was played after a correct response and a low-pitched sound was played after an incorrect response. Participants performed 16 blocks with 1280 trials in total (4 target types × 2 flanker conditions × 4 chevron distances × 20 trials per block × 2 block repetitions). The order of blocks was randomized in the first run and reversed in the second run. Subjects were instructed to respond as fast and accurately as possible. Before the start of the experiment, observers did a training session with unflanked targets. As many blocks as needed to reach on average 95% correct or higher were performed (range: 2–6 blocks). In the training, all four targets and chevron distances were intermixed in a block to provide equal exposure.

#### Data analysis

Subjects’ responses were analyzed in a mixed effects logistic regression model using R^47^ and the *lme4*^48^ package. Binary responses (correct or incorrect) were entered in the model as a dependent variable. Target type (4 levels: Diamond, X, Up-Up, and Down-Down), flanker condition (2 levels: X and Diamond), and the chevron distance (4 levels: 0.7°, 0.9°, 1.2°, and 1.5°) were entered as fixed effects. Subjects were entered as a random effect. Trials with response delays longer than 3 seconds were excluded from the analysis (58 trials in total, 0.86% of all trials). First, the model was compared to the null model (i.e., the model with no predictors) to establish whether adding independent variables improved the fit of the model. Next, the predictors of the model were assessed with the *anova* function from the *car*^49^ package. Multiple comparisons were done with the *emmeans*^50^ package, using the Tukey correction method.

### Results

**Figure 2:**
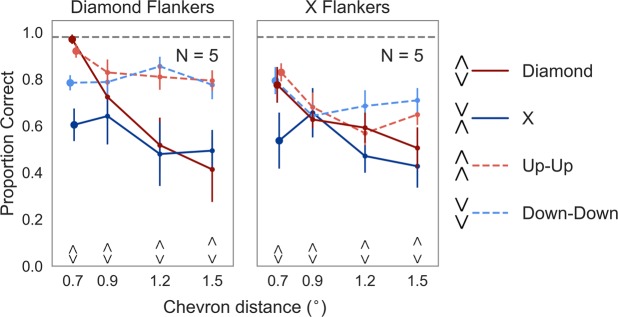
Results of Experiment 1. Average accuracy (proportion correct) as a function of center-to-center distance between the target chevrons (in degrees). The left panel shows the results for the Diamond flanker condition, the right panel for the X flanker condition. The gray dashed lines indicate the average of the unflanked condition (averaged over all targets and chevron distances). Error bars indicate standard errors of the mean. (A small horizontal jitter was added to the data points at the smallest spacing to reduce overlap of the error bars).

Figure 2 shows mean accuracy as a function of chevron distance. Adding predictors improved the model fit compared to the null model (χ(31) = 671.77, *p* < 0.001). Similarly, target type (χ(3) = 84.35, *p* < 0.001), flanker condition (χ(1) = 24.84, *p* < 0.001), and chevron distance (χ(3) = 102.17, *p* < 0.001), as well as interactions between them improved the fit (flanker condition × target type: χ(3) = 22.47, *p* < 0.001; flanker condition × chevron distance: χ(3) = 34.85, *p* < 0.001; target type × chevron distance: χ(9) = 86.57, *p* < 0.001; flanker condition × target type × chevron distance: χ(9) = 46.77, *p* < 0.001). Performance with the Diamond flankers (mean proportion correct = 0.71, SE_M_ = 0.05) was better than with the X flankers overall (mean proportion correct = 0.64, SE_M_ = 0.04; odds ratio = 1.69, SE = 0.11, *p* < 0.001), and at each distance between the chevrons (all *p*s < 0.05). In both flanker conditions, observers’ performance decreased with increasing chevron distance.

To quantify the difference between the Diamond and X targets flanked by Diamonds and Xs, we tested whether there was a difference between the two HS conditions. At the closest spacing, performance was better for Diamond targets flanked by Diamonds (mean proportion correct = 0.97, SE_M_ = 0.02), compared to X targets flanked by Xs (proportion correct = 0.54, SE_M_ = 0.12; odds ratio = 0.03, SE = 0.02, *p* < 0.001). There was no difference between the two HS conditions at larger chevron distances (all *p* > 0.8). We then compared the difference between the X and Diamond targets within each flanker condition. In the X flanker condition, performance was higher with the Diamond targets (mean proportion correct = 0.78, SE_M_ = 0.08) compared to the X targets at the closest spacing (mean proportion correct = 0.54, SE_M_ = 0.12; odds ratio = 0.32, SE = 0.07, *p* < 0.001). The two conditions did not differ at larger chevron distances (all *p* > 0.2). In the Diamond flanker condition, performance was also higher with the Diamond targets (mean proportion correct = 0.97, SE_M_ = 0.02) compared to the X targets at the closest spacing (mean proportion correct = 0.60, SE_M_ = 0.07; odds ratio = 0.04, SE = 0.02, *p* < 0.001). There was no difference between the two conditions at the other chevron distances (all *p* > 0.6). Performance with the Diamond targets flanked by Diamonds was almost perfect (mean proportion correct = 0.97, SE_M_ = 0.02), and clearly better compared to the Diamond targets flanked by Xs (mean proportion correct = 0.78, SE_M_ = 0.08; *p* < 0.001). Performance with the X targets did not differ between the two flanker conditions (Diamond flankers: mean proportion correct = 0.60, SE_M_ = 0.07; X flankers: mean proportion correct = 0.54, SE_M_ = 0.12; *p* > 0.8).

Our main analysis compared Diamond and X targets (see above). Comparing the Up-Up and Down-Down targets revealed similar performance over the tested spacings except for the closest spacing. At the closest spacing, performance with the Up-Up targets (mean proportion correct = 0.92, SE_M_ = 0.03) was better than with the Down-Down targets (mean proportion correct = 0.78, SE_M_ = 0.03; odds ratio = 3.28, SE = 1.04, *p* < 0.01) when the targets were flanked by Diamonds. Performance did not differ with the X flankers or when chevrons were positioned further apart. Interestingly, performance of both configurations remained high with increasing spacings when flanked by Diamonds.

To summarize, target type, flanker condition, and chevron distance influenced performance. Overall, performance was worse with the Xs compared to Diamonds, both when they were targets and when they were flankers. Our results showed that at the closest spacing the performance with the Diamonds flanked by Diamonds was better compared to the X targets flanked by Diamonds, showing an inversion of the target-flanker similarity rule of crowding.

## Experiment 2: Conspicuity Ratings

The results of Experiment 1 showed that at the closest spacing, Diamond targets were identified better than X targets when surrounded by the Diamond flankers. Following the target-flanker similarity rule, accuracy for targets presented among similar flankers should have been worse compared to flankers that were different. Hence, the results of Experiment 1 were in contrast to what is normally observed when the targets and the flankers are highly similar. As better performance usually goes hand in hand with higher target conspicuity, one possible explanation is that the Diamond targets stood out more from the flankers than the X targets. To test this hypothesis, we asked observers to rate the conspicuity of the target. If Diamond targets stood out more from Diamond flankers than X targets from X flankers, it could indicate that the results of Experiment 1 were driven by the higher conspicuity of Diamond targets flanked by Diamonds.

### Methods

#### Observers

Five observers participated in Experiment 2 (all females; age range = 20–25). Two of the observers had participated in the previous experiment. All participants were unaware about the purpose and the hypothesis of the experiment.

#### Apparatus, stimuli, procedure

The procedure in Experiment 2 was as in Experiment 1 with the following changes. Prior to the experiment the observers were familiarized with the concept of conspicuity (“standing out”). After each stimulus presentation, observers were asked to rate how much the target stood out from the flankers. The observers provided the responses on a 7-item Likert-type scale with 1 being the lowest rating (“Did not stand out at all”) and 7 being the highest (“Stood out strongly”). Observers pressed the corresponding key on the keyboard to record the response. No feedback was given. Observers completed 640 trials in total (4 targets × 2 flanker conditions × 4 chevron distances × 10 trials per block × 2 block repetitions).

#### Data analysis

A repeated measures ANOVA with target type (4 levels: Diamond, X, Up-Up, and Down-Down), flanker condition (2 levels: X, Diamonds), and chevron distance (4 levels: 0.7°, 0.9°, 1.2°, and 1.5°) as factors was used to analyze average conspicuity ratings. Greenhouse-Geisser corrections were applied where appropriate. Planned comparisons were tested using the non-parametric t-tests.

### Results

**Figure 3:**
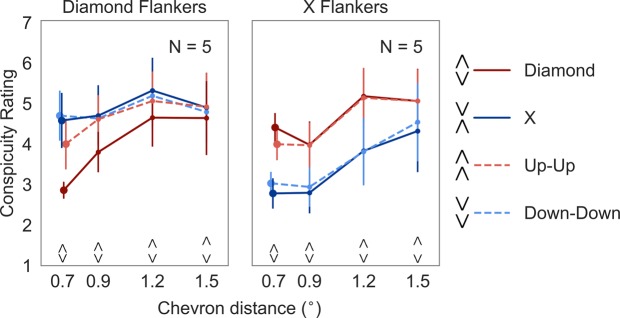
Results of Experiment 2. Average conspicuity ratings for each target in the Diamond flanker (left panel) and X flanker condition (right panel). The task was to rate the degree to which the target stood out from the flankers. Error bars denote standard error of the mean. (A small horizontal jitter was added to the data points at the smallest spacing to reduce overlap of the error bars).

Figure 3A shows the average ratings for each target type, flanker condition, and chevron distance. There was a trend for a main effect of target type (*F*(1.3, 5.2) = 5.55, *p* = 0.059, η^2^ = 0.58), with the X targets (average rating = 4.15, SE_M_ = 0.57) having a lower average rating compared to the Down-Down targets (average rating = 4.20, SE_M_ = 0.55). There was no main effect of flanker condition (*F*(1, 4) = 2.26, *p* = 0.21, η^2^ = 0.36), and no main effect of chevron distance (*F*(3, 12) = 2.03, *p* = 0.16, η^2^ = 0.34). The analysis revealed interactions between target type and flanker condition (*F*(1.057, 4.229) = 7.355, *p* = 0.05, η^2^ = 0.648), flanker condition and chevron distance (*F*(3, 12) = 6.1, *p* = 0.009, η^2^ = 0.604), and a three-way interaction between target type, flanker condition, and chevron distance (*F*(9, 36) = 3.298, *p* = 0.005, η^2^ = 0.452). Planned comparisons were performed only for the closest chevron distance since it was the spacing of interest identified in Experiment 1. We did find a standard target-flanker similarity effect: in both flanker conditions, target conspicuity was rated higher in the LS compared to the HS conditions (both *p* < 0.05). The ratings for the two HS condition did not differ: the Diamond targets flanked by Diamonds (average rating = 2.86, SE_M_ = 0.21) were not different than the X targets flanked by Xs (average rating = 2.78, SE_M_ = 0.38; Wilcoxon *Z*(4) = 0.135, *p* = 0.89). Similarly, there was no difference between the ratings in the two LS conditions: the Diamond targets flanked by Xs (average rating = 4.41, SE_M_ = 0.36) were not different than the X targets (average rating = 4.58, SE_M_ = 0.68) flanked by Diamonds (Wilcoxon *Z*(4) = 0.405, *p* = 0.686).

Ratings for the Down-Down targets closely resembled the ratings for the X targets. The ratings for the Up-Up targets were similar to the ratings for the Diamond targets, however, the ratings for the Diamond targets (average rating = 4.41, SE_M_ = 0.36) were higher than the ratings for the Up-Up targets (average rating = 3.99, SE_M_ = 0.40) when they were flanked by the X flankers (Wilcoxon *Z*(4) = 2.02, *p* = 0.04).

To sum up, X and Diamond targets were rated to stand out more in the LS compared to the HS condition, showing a standard target-flanker similarity effect. The results suggest that the higher performance when the Diamond targets were flanked by Diamonds in Experiment 1 was not due to the Diamond target’s conspicuity.

## Experiment 3: Uniformity Judgments

Experiment 1 showed a strong advantage for Diamond compared to X targets independent of the flanker type, breaking the similarity rule of crowding. In Experiment 2, we found that target conspicuity ratings were in line with the similarity rule, showing that target conspicuity is an unlikely cause for the Diamond advantage in Experiment 1. To investigate if another potential source of target information – display uniformity – could have driven the results of Experiment 1, we investigated perceived uniformity of the displays with Diamond and X flankers. Observers were asked to report whether all items (i.e., the target and the flankers) in the display were the same or not. If uniformity judgments were superior with all Diamonds compared to all Xs, uniformity would be a possible contributing factor to the Diamond advantage observed in Experiment 1.

### Methods

#### Observers

Five observers (4 females, 1 male; age range = 22–36) participated in the experiment. One observer had participated in Experiment 1, and another observer in Experiments 1 and 2. All participants were unaware about the purpose and the hypothesis of the experiment.

#### Apparatus, stimuli, procedure

The procedure in Experiment 3 was as in Experiment 1 but with the following changes. After the presentation of the target, observers reported whether all items (i.e., the target and the flankers) were the same or not by pressing a key associated with’yes’ (all items were the same) or’no’ (not all items were the same). For example, when the Diamond was flanked by Diamonds (or X was flanked by Xs) observers were required to respond with ‘Yes, all items were the same’ and when the X was flanked by Diamonds, observers were required to respond with ‘No, the items were not the same’. Only the closest spacing between the chevrons was used in this experiment (center-to-center spacing = 0.7°). No feedback was given.

#### Data analysis

The responses were analyzed in a mixed effects logistic regression model using R^47^ and the *lme4*^48^ package. The procedure followed the one used in Experiment 1. Binary responses (correct or incorrect) were entered in the model as a dependent variable. Target type (4 levels: Up-Up, Down-Down, Diamond, and X), and flanker condition (2 levels: X and Diamond) were entered as fixed effects. Subjects were entered as a random effect.

### Results

**Figure 4:**
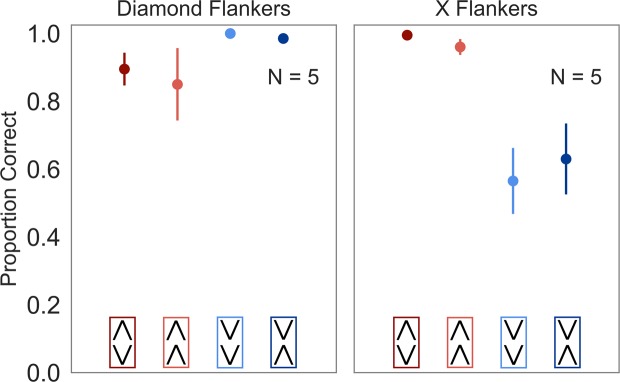
Results of Experiment 3. Average uniformity judgments for each target when flanked by the Diamonds (left panel) and Xs (right panel) at the smallest chevron distance (see text for details). Error bars denote standard error of the mean. A horizontal jitter was added to reduce overlap of the error bars.

Figure 4 shows averaged accuracies of uniformity judgments. Inclusion of predictors improved the model fit compared to the null model (χ(7) = 355.52, *p* < 0.001). Target type (χ(3) = 16.86, *p* < 0.001), flanker condition (χ(1) = 9.62, *p* < 0.01), and an interaction between the predictors (χ(3) = 61.63, *p* < 0.001) improved the fit of the model. Observers’ judgments differed between the two HS conditions; they were more accurate when the Diamond targets were flanked by Diamonds (average proportion correct = 0.90, SE_M_ = 0.05) compared to X targets flanked by Xs (average proportion correct = 0.63, SE_M_ = 0.10; odds ratio = 0.18, SE = 0.05, *p* < 0.001). There were no differences in accuracy in the LS condition (p < 0.9): judgments for the Diamond targets flanked by Xs (average proportion correct = 0.99, SE_M_ = 0.01) and the X targets flanked by Diamonds (average proportion correct = 0.98, SE_M_ = 0.01) were both highly accurate.

Identifying the orientation of the upper target chevron in the conditions in which its orientation was opposite of that required for all items being the same was sufficient to correctly indicate the absence of uniformity (if the type of flankers was identified). The high performance in all conditions in which this was the case (all above 96%; X and Down-Down targets flanked by Diamonds; Diamond and Up-Up targets flanked by Xs) supports the notion that a *discrepant* orientation of the upper target chevron could be used to perform the task. However, its orientation was not useful to differentiate between Diamond and Up-Up targets flanked by Diamonds, and X and Down-Down targets flanked by Xs, i.e., where it was in line with all items being the same. Hence, we compared performance for Up-Up targets flanked by Diamonds with that of Down-Down targets flanked by Xs. When flanked by Xs, accuracy for Down-Down targets (average proportion correct = 0.57, SE_M_ = 0.10) was worse than the accuracy for Up-Up targets (average proportion correct = 0.85, SE_M_ = 0.11; odds ratio = 0.21, SE = 0.02, *p* < 0.001) flanked by Diamonds, indicating that there was a benefit for Up-Up targets driven by the alternative with the *same* upper chevron (Diamond targets) in the Diamond flanker condition.

Taken together, Experiment 3 showed that while observers’ uniformity judgments were similar and highly accurate in the LS conditions, the pattern of judgments in the HS conditions depended on the flanker type. Observers judged the uniformity of the stimulus more accurately when Diamond targets were flanked by Diamonds than when X targets were flanked by Xs. Within each flanker condition, accuracy was similar when the upper target chevron was the same.

## Discussion

In ecologically valid situations, task-relevant information can usually be extracted from various sources. In most crowding studies, however, the flankers and target-flanker relations do not provide information about target identity. Here, we investigated if the similarity rule of crowding (i.e., higher target-flanker similarity yielding stronger crowding) held when additional information on different levels of perceptual organization was available in the stimuli.

In Experiment 1, we observed that identification performance was higher when Diamonds were flanked by Diamonds compared to Xs flanked by Diamonds, violating the similarity rule of crowding. Experiment 2 showed that this Diamond advantage cannot be explained by target conspicuity as the standard target-flanker similarity effect was observed when observers rated the conspicuity of the targets: Diamonds were judged to stand out more from X flankers than from Diamond flankers, and X targets were judged to stand out more from Diamond flankers than from X flankers. In Experiment 3, we demonstrated that uniformity judgments were more accurate when Diamonds were flanked by Diamonds compared to Xs flanked by Xs. We suggest that the uniformity of the displayed items contributed to the overall superior performance with Diamonds compared to Xs.

Our results show that figural characteristics of the Diamonds and the Xs determined performance. While Diamonds and Xs were formed by the same chevrons, they differed in figural complexity and figural qualities (good Gestalt and topological properties, see below). For example, X flankers were more complex than Diamond flankers in regard to perimetric complexity^51,52^, skeleton length^16^, and number of turns^53,54^. Hence, flanker complexity alone – with more complex flankers yielding stronger crowding than less complex flankers^55^ – could explain the overall low performance with X compared to Diamond flankers. However, flanker complexity cannot explain the high performance with Diamond compared to X targets when the flankers were Diamonds. Rather, we suggest that the emergent good Gestalt of the Diamonds compared to that of the Xs provided an advantage, yielding better performance with Diamond than X targets. Such an advantage of Diamond’s compared to other targets’ emergent features^31,56^ was already shown by Pomerantz and Garner^57^ who informally collected goodness ratings on stimuli consisting of two parentheses and found that configurations grouped into circle-like configurations (similar to the Diamonds in the present study) were perceived as ‘better’ compared to X-like configurations (similar to the Xs). These results anticipated the now often shown superiority of closed compared to open shapes (see e.g.^19,20,29,58–60^). In crowding closed configurations flanking a vernier target interfered less with target perception than ‘open’ (scrambled) configurations consisting of the same elements^20^. Similarly, flankers that contained an X-configuration deteriorated performance less strongly when the X was surrounded by a closed rectangle^19^. Moreover, in a similar paradigm as in the present study, we showed an advantage for Diamond compared to X targets with ‘neutral’ flankers (consisting of single chevrons^29^). Note that neither the uniqueness of closure among the four targets nor a general advantage for Diamonds independent of crowding underlies the present findings: although closure was a unique feature of the Diamonds, other unique features such as the X-junction or collinearity of the Xs did not prevent the Diamond advantage. As there was no advantage for unflanked Diamonds compared to unflanked Xs (performance for all four unflanked target configurations was above 95% correct), it can be excluded that the difference in performance was due to an unequal ability to discriminate the targets. Moreover, as the Diamonds and Xs consisted of the same parts, the Diamond advantage can also not be reduced to its constituent parts but is genuinely due to its figural quality which seems to be strong enough to reverse the similarity rule of crowding (see below for the role of perceived uniformity as a potential source for the Diamond advantage). The targets (and flankers) also differed in their topological properties (any properties that are preserved under all one-to-one, continuous transformations^61^). For example, the Diamonds as objects ‘with holes’ were topologically different from the Xs. The processing of such global topological features has been proposed to precede the processing of other features^61^, and topological differences were suggested to play a key role in the emergence of configural superiority effects^62^. Hence, topological differences between the different target (and flanker) configurations – as well as figural quality in general – may well play a role in our results.

Importantly, this figural quality appears to be unrelated to conspicuity. Usually, performance is strongly related to the conspicuity of the target: when the target is conspicuous, i.e., it stands out from the flankers, crowding is weaker than when the target is not conspicuous^21,24,26^. Hence, better performance with the Diamond targets in Experiment 1 would be expected to be associated with higher target conspicuity. In Experiment 2, observers rated the degree to which the target stood out from the flankers (conspicuity ratings). If Diamond targets stood out more from Diamond flankers than X targets from X flankers, the results of Experiment 1 could have been driven by target conspicuity. However, if both targets stood out similarly, target conspicuity would not explain the results of Experiment 1. The results of Experiment 2 showed a typical target-flanker similarity effect: Diamonds and Xs were judged to stand out more from different than from similar flankers. Thus, the performance differences between Diamonds and Xs in Experiment 1 (and thereby the violation of the target-flanker similarity rule of crowding) were not due to differences of target conspicuity.

Instead, we suggest that display uniformity^63^ provided information that could be used by observers to perform the task when Diamond targets were presented among Diamond flankers, but not when X targets were presented among X flankers. Perceiving a cluster of target and flankers as uniform may improve performance when the target is the same as the flankers because identifying one item of the cluster is sufficient to respond correctly^41,45^. In Experiment 3, we asked observers to judge the uniformity of the display by indicating whether all displayed elements (target and flankers) were the same or not. Similar to Experiment 1, there was a marked Diamond advantage: uniformity judgments for Diamond targets flanked by Diamonds were more accurate than for X targets flanked by Xs. Interestingly, performance for all targets was relatively high with Diamond flankers where both presence and absence of uniformity was accurately reported. By contrast, performance with X flankers was high for Diamond and Up-Up targets and low for X and Down-Down targets. The low performance for X targets flanked by Xs (63% (SE_M_ = 10.5) correct, vs. 89.5% (SE_M_ = 4.5) correct for Diamonds among Diamonds) was associated with participants’ inability to distinguish them from Down-Down targets: in 43.5% of the trials with Down-Down targets the displays were judged as uniform (in comparison, only 15% of the trials with Up-Up targets were erroneously judged as the same as the flankers in the Diamond flanker condition). Similar to Experiment 1, these results showed that targets with different orientations of the upper chevron were rarely confused with each other (confusions at the smallest spacing in Experiment 1 with different upper chevron: 2.2% (SE_M_ = 0.76)); with the same upper chevron: 18% (SE_M_ = 2.6)). While the results of Experiment 3 do not ensure that display uniformity helped participants in Experiment 1, they indicate that uniformity could serve as a cue to successfully perform the task, however, only when all items were Diamonds.

Taken together, the present results cannot be explained by interference between neighboring chevrons alone. Rather, the perceptual organization of the displayed items on different levels needs to be considered to explain performance^58,64^. In Experiment 1, the grouping of the two target chevrons was necessary to perform the identification task. In line with studies showing that grouping may yield both detrimental^15,19,24^ and advantageous effects in crowding^28,29^ depending on whether it hinders or improves access to the target’s features, the strong dependence of performance on the different targets in the present study (in particular Diamonds compared to Xs) showed that a combination of the two target chevrons independent of their configuration cannot underlie our findings. Rather, the specific grouping of the chevrons and their emergent features need to be taken into consideration to explain the pattern of results. The different groupings of the two target chevrons alone, however, do not explain the violation of the target-flanker similarity rule: as identification of Diamonds was superior than identification of Xs, and conspicuity ratings of Diamonds were lower compared to Xs (when flanked by Xs), it seems that a factor different from conspicuity contributed to the Diamond advantage. We propose that this factor was display uniformity.

Similar to superior performance in natural environments when stimulus relations are informative^33,35^, our results showed that information available on different levels of perceptual organization can be useful for task completion in a crowding paradigm. Emergent features of the target and the uniformity of the display were sufficiently potent cues to overrule the similarity rule of crowding. We suggest that conventional crowding rules only hold when the relations between the elements in the display are not informative.

## Data Availability

The datasets generated during and/or analysed during the current study are available from the corresponding author on reasonable request.

## References

[1] Bouma H (1970). Interaction Effects in Parafoveal Letter Recognition. Nature.

[2] Bouma H (1973). Visual interference in the parafoveal recognition of initial and final letters of words. Vision Res..

[3] Pelli DG, Tillman KA (2008). The uncrowded window of object recognition. Nat. Neurosci..

[4] Whitney D, Levi DM (2011). Visual crowding: a fundamental limit on conscious perception and object recognition. Trends Cogn. Sci..

[5] Coates Daniel R., Wagemans Johan, Sayim Bilge (2017). Diagnosing the Periphery: Using the Rey–Osterrieth Complex Figure Drawing Test to Characterize Peripheral Visual Function. i-Perception.

[6] Sayim B, Wagemans J (2017). Appearance changes and error characteristics in crowding revealed by drawings. J. Vis..

[7] Greenwood John A., Bex Peter J., Dakin Steven C. (2010). Crowding Changes Appearance. Current Biology.

[8] Toet A, Levi DM (1992). The two-dimensional shape of spatial interaction zones in the parafovea. Vision Res..

[9] Kooi FL, Toet A, Tripathy SP, Levi DM (1994). The effect of similarity and duration on spatial interaction in peripheral vision. Spat. Vis..

[10] Manassi M, Sayim B, Herzog MH (2013). When crowding of crowding leads to uncrowding. J. Vis..

[11] Põder E (2007). Effect of colour pop-out on the recognition of letters in crowding conditions. Psychol. Res..

[12] Chung STL, Mansfield JS (2009). Contrast polarity differences reduce crowding but do not benefit reading performance in peripheral vision. Vision Res..

[13] Nazir TA (1992). Effects of lateral masking and spatial precueing on gap-resolution in central and peripheral vision. Vision Res..

[14] Zahabi S, Arguin M (2014). A crowdful of letters: Disentangling the role of similarity, eccentricity and spatial frequencies in letter crowding. Vision Res..

[15] Sayim B, Westheimer G, Herzog MH (2008). Contrast polarity, chromaticity, and stereoscopic depth modulate contextual interactions in vernier acuity. J. Vis..

[16] Bernard J-B, Chung STL (2011). The dependence of crowding on flanker complexity and target-flanker similarity. J. Vis..

[17] Banks WP, Larson DW, Prinzmetal W (1979). Asymmetry of visual interference. Percept. Psychophys..

[18] Malania M, Herzog MH, Westheimer G (2007). Grouping of contextual elements that affect vernier thresholds. J. Vis..

[19] Manassi M, Sayim B, Herzog MH (2012). Grouping, pooling, and when bigger is better in visual crowding. J. Vis..

[20] Sayim B, Westheimer G, Herzog MH (2010). Gestalt Factors Modulate Basic Spatial Vision. Psychol. Sci..

[21] Sayim B, Westheimer G, Herzog MH (2011). Quantifying target conspicuity in contextual modulation by visual search. J. Vis..

[22] Livne T, Sagi D (2007). Configuration influence on crowding. J. Vis..

[23] Livne T, Sagi D (2010). How do flankers’ relations affect crowding?. J. Vis..

[24] Saarela TP, Sayim B, Westheimer G, Herzog MH (2009). Global stimulus configuration modulates crowding. J. Vis..

[25] Sayim, Cavanagh P (2013). Grouping and Crowding Affect Target Appearance over Different Spatial Scales. PLoS ONE.

[26] Saarela TP, Westheimer G, Herzog MH (2010). The effect of spacing regularity on visual crowding. J. Vis..

[27] Felisberti FM, Solomon JA, Morgan MJ (2005). The Role of Target Salience in Crowding. Perception.

[28] Sayim B, Greenwood JA, Cavanagh P (2014). Foveal target repetitions reduce crowding. J. Vis..

[29] Melnik N, Coates DR, Sayim B (2018). Emergent features in the crowding zone: When target–flanker grouping surmounts crowding. J. Vis..

[30] Pomerantz J, Cragin A (2012). Crowding, Grouping, and the Configural Superiority Effect. J. Vis..

[31] Pomerantz J, Sager LC, Stoever RJ (1977). Perception of wholes and of their component parts: some configural superiority effects. J. Exp. Psychol. Hum. Percept. Perform..

[32] Weisstein N, Harris CS (1974). Visual Detection of Line Segments: An Object-Superiority Effect. Science.

[33] Biederman I, Mezzanotte RJ, Rabinowitz JC (1982). Scene perception: Detecting and judging objects undergoing relational violations. Cognit. Psychol..

[34] Bar M (2004). Visual objects in context. Nat. Rev. Neurosci..

[35] Wijntjes MWA, Rosenholtz R (2018). Context mitigates crowding: Peripheral object recognition in real-world images. Cognition.

[36] Hollingworth A, Henderson JM (2000). Semantic Informativeness Mediates the Detection of Changes in Natural Scenes. Vis. Cogn..

[37] Reicher GM (1969). Perceptual recognition as a function of meaningfulness of stimulus material. J. Exp. Psychol..

[38] Torralba A (2003). Contextual Priming for Object Detection. Int. J. Comput. Vis..

[39] Oliva A, Torralba A (2007). The role of context in object recognition. Trends Cogn. Sci..

[40] Geisler WS, Perry JS, Super BJ, Gallogly DP (2001). Edge co-occurrence in natural images predicts contour grouping performance. Vision Res..

[41] Taylor H, Sayim B (2018). Crowding, attention and consciousness: In support of the inference hypothesis. Mind Lang..

[42] Nickerson RS (1965). Response Times for Same-Different Judgments. Percept. Mot. Skills.

[43] Bindra D, Donderi DC, Nishisato S (1968). Decision latencies of “same” and “different” judgments. Percept. Psychophys..

[44] Young ME, Wasserman EA (2002). Detecting variety: What’s so special about uniformity?. J. Exp. Psychol. Gen..

[45] Sayim, B. & Taylor, H. Letters Lost: Capturing Appearance in Crowded Peripheral Vision Reveals a New Kind of Masking. *Psychol. Sci*. 095679761984716 (2019). 10.1177/0956797619847166 31120814

[46] Peirce JW (2007). PsychoPy—Psychophysics software in Python. J. Neurosci. Methods.

[47] R Core Team. R: A language and environment for statistical computing. R Foundation for Statistical Computing Vienna, Austria. https://www.R-project.org/ (2017).

[48] Bates, D., Mächler, M., Bolker, B. & Walker, S. Fitting Linear Mixed-Effects Models Using lme4. *J. Stat. Softw*. **67**, 10.18637/jss.v067.i01 (2015).

[49] Fox, J. & Weisberg, S. *An R companion to applied regression*. (Sage Publications, Inc, 2019).

[50] Lenth, R. *Emmeans: estimated marginal means, aka least-squares means*. (2018).

[51] Attneave F, Arnoult MD (1956). The quantitative study of shape and pattern perception. Psychol. Bull..

[52] Pelli DG, Burns CW, Farell B, Moore-Page DC (2006). Feature detection and letter identification. Vision Res..

[53] Arnoult MD (1960). Prediction of perceptual responses from structural characteristics of the stimulus. Percept. Mot. Skills.

[54] Attneave F (1957). Physical determinants of the judged complexity of shapes. J. Exp. Psychol..

[55] Zhang J-Y, Zhang T, Xue F, Liu L, Yu C (2009). Legibility of Chinese characters in peripheral vision and the top-down influences on crowding. Vision Res..

[56] Pomerantz, J. R. & Cragin, A. I. Emergent features and feature combination in *The Oxford handbook of perceptual organization* (ed. Wagemans, J.) 88–107 (2015).

[57] Pomerantz JR, Garner WR (1973). Stimules configuration in selective attention tasks. Percept. Psychophys..

[58] Pirkner Y, Kimchi R (2017). Crowding and perceptual organization: Target’s objecthood influences the relative strength of part-level and configural-level crowding. J. Vis..

[59] Elder J, Zucker S (1993). The effect of contour closure on the rapid discrimination of two-dimensional shapes. Vision Res..

[60] Meng Q (2018). The dissociations of visual processing of “hole” and “no-hole” stimuli: An functional magnetic resonance imaging study. Brain Behav..

[61] Chen L (2005). The topological approach to perceptual organization. Vis. Cogn..

[62] Pomerantz J (2003). Wholes, holes, and basic features in vision. Trends Cogn. Sci..

[63] Donderi DC, Zelnicker D (1969). Parallel processing in visual same-different decisions. Percept. Psychophys..

[64] Herzog MH, Sayim B, Chicherov V, Manassi M (2015). Crowding, grouping, and object recognition: A matter of appearance. J. Vis..

